# A rare case of paralysis in an endemic area

**DOI:** 10.12669/pjms.312.7045

**Published:** 2015

**Authors:** Bulent Yardimci, Rumeyza Kazancioglu

**Affiliations:** 1 Bulent Yardimci, MD. Department of Internal Medicine, Istanbul Florence Nigtingale Hospital, Istanbul, Turkey; 2 Rumeyza Kazancioglu, MD. Professor Doctor, Department of Nephrology, Bezmialem Vakif University, Faculty of Medicine, Istanbul, Turkey

**Keywords:** Thyrotoxicosis, Hypokalemia, Hypokalemic Periodic Paralysis, Periodic Paralysis

## Abstract

Thyrotoxicosis mostly presents with tachycardia, tremor, weight loss and other hypermetabolism signs. However, there are other unusual signs of thyrotoxicosis such as paralysis. This unusual clinical presentation may postpone prompt diagnosis and treatment. In this case report, we present a 27-years-old woman, who presented with quadriparesis at the emergency department.

## INTRODUCTION

Thyrotoxic Periodic Paralysis (TPP) is a rare complication of hyperthyroidism, which mainly affects Asian men.^1^-^3^ The diagnosis is usually missed because it is a rare presentation of thyrotoxicosis. Therefore, a proper treatment is often delayed. The first step in diagnosis is to have a thorough knowledge concerning thyrotoxicosis and its complications. TPP should be highly suspected in patients, who present with a quadriparesis, hypokalemia and dysrhythmia.^1^ Prompt diagnosis is of utmost importance because it may be fatal unless treated.^1^ Herein, we present a young woman, who presented with thyrotoxicosis and generalised paralysis.

## CASE REPORT

A 27 years-old female presented at the emergency department with complaints of nausea, vomiting and inability to move all extremities which lasted for six hours. She had sore throat seven days ago and used amoxycillin-clavulanate tablet (tab) 2×1000 mg for five days. Her past medical history revealed quadryparesis attacks for the last 5 months. These attacks mostly came at night with cramps and weakness, which lasted about 2-3 hours. The frequency of the attacks was about every two weeks. She was diagnosed hyperthyroidism two months ago and she was put on propylthiouracil tab. 75 mg/day and propranolol tab. 40 mg/day.

Physical examination revealed that she was fully conscious, well oriented. Her blood pressure was 95/50 mmHg, heart rate was 110/min rhythmic and body temperature was 37ºC. Her muscle strength was 2/5 in lower and 3/5 upper extremities. The deep tendon reflexes were decreased.

Her laboratory results are presented in Table-I. The levels of hemoglobin, hematocrit, thrombocytes, bilirubins, creatinine, uric acid, calcium, magnesium, sodium, chloride and urinalysis were within normal limits. Thyroid function tests showed TSH as 0.005 uU/mL, free T4 as 4.98 ng/dL and free T3 as 15.54 pg/ml (Table-II) Electrocardiography showed sinusal tachycardia, abdominal ultrasonography showed a gallstone of 0.5 cm in diameter, and normal gallbladder wall. Chest X-ray was normal.

**Table I:** The laboratory results of the patient.

Serum		Patient initial result	Normal range
Potassium	mmol/L	1.5	3.5-5.5
ALT	U/L	159	<31
AST	U/L	107	<31
BUN	mg/dL	30.8	5-23
Glucose	mg/dL	155	70-110
hsCRP	mg/L	57.2	< 5
WBC	mm3/L	15740	4100-11000

**Table II:** Thyroid function test of patient.

Serum		Patient initial result	Normal range
TSH	uU/mL	0.005	0.27- 4.20
Free T3	pg/mL	15.53	1.80-4.60
Free T4	g/dL	4.98	0.90-1.70

She was admitted to the intensive care unit. She was administered potassium chloride with the infusion rate of 10 mEq/h, followed by oral potassium supplement on day two. Therapy for hyperthyroidism was rearranged as propylthiouracil 3X100 mg and propranolol 2X40 mg/day. On the second day, her pulse was 80/min regular and potassium level was 2,6mmol/L. On the third day her muscle strength was 3/5 lower and 4/5 upper extremities, and potassium level was 3.1 mmol/L. Fifth day her muscle strength was 5/5 lower and upper extremities. Her liver enzymes and hs-CRP returned back to normal values in four days. She was discharged six days later with normal muscle strength and potassium level of 3.8 mmol/L.

## DISCUSSION

Rapid onset of paralysis is a frightening condition for the patients and their relatives, especially in the young. Possible causes of this condition include Guillian-Barré Syndrome, Spinal Cord Compression, Familial Periodic Paralysis or Sporadic Paralysis and TPP which is are complications of thyrotoxicosis.^1^, ^2^

TPP is mostly prevalent among Asian men.^1^,^3^ Men are affected nearly 17-70 fold more than women and it is mostly seen between 20-40 years of age.^3^-^7^ Furthermore the rate of TPP in North America and Europe is 0.1-0.2 % among thyrotoxic patients although it is 2% in China and Japan.^8^

The mechanism of TPP is through the sodium pump of the cells. Sodium-potassium ATPase transports the potassium into the cells. The activity and number of sodium pumps on white blood cells and muscle cells are increased in hyperthyroidism.^1^ This mechanism causes hypokalemia in TPP. Cesur et al.^9^ reported the result of 40 TPP cases in Turkey. All Turkish TPP patients were sporadic cases without any family history. Man –to-female ratio was 20:1.^9^ Our case is unique by being female without any positive family history concerning periodic paralysis. She had experienced periodic attacks for 5 months and hyperthyroidism was diagnosed 2 months ago. Laboratory tests showed that hyperthyroidism wasn’t well treated.

TPP cases usually present at the emergency department following carbohydrate- rich meal, heavy exercise or with any other precipitant factors such as alcohol use, upper respiratory tract infection, emotional stress etc. Our patient also had a sore throat ongoing for the last 7 days. She had gallbladder stone, but there was not any sign of gallbladder inflammation. This finding may confirm the presence of an inciting factor before a paralysis effect.

Although hyperthyroidism may cause sinus tachycardia, QT prolongation, atrial and ventricular dysrhythmias, we detected only sinusal tachycardia and it was corrected with potassium infusion and effective beta blockage.^1^,^3^

## CONCLUSION

Sudden onset of paralysis is frequently associated with neurological diseases in emergency departments. However, in endemic areas such as Turkey, TPP should also be suspected, when it is accompanied with hypokalemia.
